# Targeting nucleocytoplasmic transport in cancer therapy

**DOI:** 10.18632/oncotarget.1457

**Published:** 2013-11-24

**Authors:** Richard Hill, Bastien Cautain, Nuria de Pedro, Wolfgang Link

**Affiliations:** ^1^ Regenerative Medicine Program, Departamento de Ciências Biomédicas e Medicina, Universidade do Algarve, Portugal; ^2^ IBB-Institute for Biotechnology and Bioengineering, Centro de Biomedicina Molecular e Estrutural, Universidade do Algarve, Campus de Gambelas, Faro, Portugal; ^3^ Fundacion MEDINA Parque tecnológico ciencias de la salud, Avd. Conocimiento 3, Granada, Spain

**Keywords:** Tumor suppressors, Cancer, Anti-cancer therapy, Nuclear export, nuclear import

## Abstract

The intracellular location and regulation of proteins within each cell is critically important and is typically deregulated in disease especially cancer. The clinical hypothesis for inhibiting the nucleo-cytoplasmic transport is based on the dependence of certain key proteins within malignant cells. This includes a host of well-characterized tumor suppressor and oncoproteins that require specifc localization for their function. This aberrant localization of tumour suppressors and oncoproteins results in their their respective inactivation or over-activation. This incorrect localization occurs actively via the nuclear pore complex that spans the nuclear envelope and is mediated by transport receptors. Accordingly, given the signifcant need for novel, specifc disease treatments, the nuclear envelope and the nuclear transport machinery have emerged as a rational therapeutic target in oncology to restore physiological nucleus/cytoplasmic homeostasis. Recent evidence suggests that this approach might be of substantial therapeutic use. This review summarizes the mechanisms of nucleo-cytoplasmic transport, its role in cancer biology and the therapeutic potential of targeting this critical cellular process

## INTRODUCTION

The physical separation of the genome from the cytoplasm by the nuclear envelope (NE) is a hallmark of the eukaryotic cell creating a requirement to transport macromolecules across the nuclear membrane to meditate their normal function(s). This transport process is highly coordinated and is an important regulator of cell signalling (to growth signals or various stresses). The separation of proteins from their site(s) of function is a recurrent motif ensuring the fdelity of signal transduction and preventing aberrant activation. Many transcription factors reside in the cytoplasm in an inactive form until activation is triggered that results in their translocation into the nucleus. Upon nuclear entry they direct specifc transcriptional programs determining cell fate [1]. Cancer cells utilize the aberrant localization of tumor suppressor proteins as a means for their inactivation and to effectively evade anti-neoplastic therapies [2]. The aberrant localization of oncoproteins can also lead to their inadequate activation [3]. Our growing understanding of the mechanisms that control the nucleo-cytoplasmic transport has directed the development new exciting therapeutic avenues to target cancers that display aberrant protein subcellular localization.

### The nucleoplasmatic transport

Protein transport is a critical, highly regulated process ensuring that the nuclear entry of proteins through the nuclear pore complex (NPC) only occurs when their functions are required and to export them into the cytoplasm when they are not needed. For nuclear entry, proteins must negotiate the NPC that is comprised of over 30 different protein components called nucleoporins (Nups) [4]. While proteins less than 40 kDa in size are able to freely traverse the NPC, larger proteins and molecules require active transport that is directed by nuclear localization or nuclear export sequences (NLS or NES) [5]. Import is controlled by importins (either α or β sub-types). After being bound by importin, the resulting cargo protein-importin complex is brought inside the nucleus via Nups located within the NPC itself. Once this protein-protein complex has entered the nucleus, the cargo/transport protein complex is bound by Ran-GTP dissociating the transport components. This releases the cargo protein allowing it to direct its function(s) within the nucleus and enables the recycling of the transport proteins for subsequent rounds of nuclear transport. The export of proteins from the nucleus is controlled by the recognition of a leucine rich sequence (NES) within the cargo protein by CRM1/exportin1. This binds to Ran-GTP and traverses the NPC into the cytoplasm. The conversion of Ran-GTP to Ran-GDP induces the dissociation of the complex allowing the recycling of the export molecules.

### Nucleoporins and cancer

Several Nups have been linked to tumor formation and progression [6]. The nucleoporin Rae1 (Gle2) plays an important role in RNA export [7] and is linked to breast cancer pathology [8]. The over expression or the depletion of Rael1 directs the formation of multi-polar spindles and promotes aneuploidy [9]. Nup98 is also a well indentifed proto-oncogene in leukemia [10] and is also part of a recurrent chromosomal translocation event observed in acute myeloid leukemia patients [11]. TPR (translocated promoter region and part of the NPC), was named after its initial isolation from a carcinogen treated asteogenic sarcoma cell line as part of a chromosomal translocation event that fused the N-terminal sequence of TPR to the kinase domain of the proto-oncogene Met [12], an early step in the formation of gastric carcinomas [6]. Nup88 is over expressed in ovarian cancer [13], lymphomas, mesotheliomas, a broad spectrum of sarcomas and in some epithelial cancers [14] In breast, colorectal and hepatocellular carcinoma Nup88 over expression is associated with tumor aggressiveness [15].

### Nuclear Transport Receptors (NTRs) and cancer

The over expression of karyopherin-β family members is observed in colon, breast and lung cancer [16;17] while the deregulation of karyopherin-α2 expression in breast cancer and melanoma correlates with poor prognosis and reduced overall survival [16]. Elevated CRM1 expression correlates with poor clinical outcome in ovarian [18], pancreatic [19], osteosarcoma [20], glioma [21] and cervical cancers [22]. Furthermore, the truncated form of karyopherin-α (that lacks the NLS binding domain) was observed in the breast cancer cell line ZR- 75-1 [23] resulting in the accumulation of p53 in the cytoplasm. In addition to karyopherin-α truncations, CAS (a factor that regulates karyopherin-α movement) is over expressed in breast cancers [24] liver neoplasms [24;25] and colon cancer cell lines [26]. Consequently any change in the expression of CAS can signifcantly affect the nuclear transport and subcellular distribution of tumor suppressors and oncoproteins.Nucleoplamic transport of tumor suppressors and oncogenic proteins

Considering the importance of the subcellular localization of proteins it is not surprising that the disruption of nuclear-cytoplasmic transport is oncogenic and a potent mechanism for cancer cells to evade and develop resistance to chemotherapeutic treatments. This is striking as drug resistance continues to be one of the most signifcant obstacles for the effective treatment of cancer and offers the cancer cell the ability to gain resistance to a broad range of chemotherapeutic drugs whose mechanisms of action could be highly divergent. A number of the major oncogenes and tumour suppressors with aberrant subcellular localization in cancer are shown in Table 1. Proteins that exert essential functions within the nucleus to prevent cancer initiation, progression or to direct the cellular response to chemotherapeutic assault include p53, FOXO(s), p27, BCRA1, APC, nucleophosmin and retinoblastoma (Rb). In contrast, the activation and cellular mis-localization of pro-survival and proliferation proteins such as β-catenin, NF-κB, survivin or cyclin D1 have been reported in various types of cancers [2;27]. Given these observations, the proteins involved in the subcellular localization of these factors are highly attractive therapeutic targets as they are both essential while the restoration of their aberrant function(s) would not be detrimental to non-cancerous cells.

**Table 1:** Differential subcellular localization of proteins in human cancer

Protein	Function	Cancer	Normal Localization	Mislocalization	Reference
FOXO	Transcription factor	Various types of cancer	Nucleus	Cytoplasm	[141]
β-Cantenin	Wnt signaling	Colorectal cancer	Cytoplasm	Nucleus	[2]
p53	Transcription factor	Various types of cancer	Nucleus	Cytoplasm	[142]
Galectin-3	Beta-galactoside-BP	Various types of cancer	Nucleus	Cytoplasm	[141]
BARD1	BRAC1 interacting Protein	Breast cancer	Cytoplasm	Nucleus	[143]
BRAC1	DNA repair	Breast cancer	Cytoplasm	Nucleus	[64]
NF-kB	Transcription factor	Various types of cancer	Cytoplasm	Nucleus	[144]
NPM1	Ribonucleoprotein	AML	Nucleolus	Cytoplasm	[145]
p21WAF1	Cell cycle inhibitor	CML, ovarian, breast	Nucleus	Cytoplasm	[146]
p27KIP	Cell cycle inhibitor	AML, breast	Nucleus	Cytoplasm	[147]
RUNX3	Transcription factor	Gastric cancer	Nucleus	Cytoplasm	[148]
INI1	Tumor suppressor	Rhabdoid tumors	Nucleus	Cytoplasm	[149]
RB	E2F BP	Various types of cancer	Nucleus	Cytoplasm	[145]
HIF-1α	Transcription factor	Breast, prostate cancer	Cytoplasm	Nucleus	[112]
N FAT	Transcription factor	Various types of cancer	Nuc/Cyt	Nucleus	[150]
PTEN	Phosphatase	Various types of cancer	Nuc/Cyt	Cytoplasm	[151]
Bcr-Abl	Kinase	CML	Nuc/Cyt	Cytoplasm	[152]
Fbw7γ	Ubiquitin ligase	Various types of cancer	Nucleolus	not nucleolar	[153]

### p53

The tumor suppressor and transcription factor p53 is one of the most commonly mutated proteins in all human cancers indicating its vital role in genome protection [28]. The majority of these mutations target the DNA binding ability of p53 [29] although this is not always the case. In several types of cancer (including breast, colon, ovarian, retinoblastoma and neuroblastoma) the inactivation of wild-type p53 is by abnormal cytoplasmic localization [30;31]. In these situations, the functionality of the p53 protein (including its ability to bind DNA) remains intact and p53 activity is, in principle restorable by relocating the protein into the nucleus. p53 contains both NLS and NES signals that mediate nuclear import and nuclear export via importin-α/ß and CRM1. The NLS is adjacent to, and the NES is contained within, the oligomerization domain of p53 raising the possibility that oligomerization of p53 may regulate p53 nucleo-cytoplasmic transport by affecting the accessibility of the NLS and/or the NES to their respective receptors. Following cellular stress p53 is post-translationally modifed and translocates into the nucleus where it induces the transcription of its target genes driving cell cycle arrest of apoptosis [32]. In addition to directing p53 to the proteosome for degradation, p53 ubiquitination (by MDM2) also functions as a signal for degradation-independent roles that includes nuclear export. While polyubiquitinated p53 is targeted for proteasomal degradation, mono-ubiquitinated p53 is specifcally targeted for nuclear export [33;34] and directly contributes to the cellular choice between cell cycle arrest, senescence or apoptosis.

The cytoplasmic sequestration of p53 in neuroblastoma typically results from a MDM2 amplifcation event and subsequent mono-ubiquitination [35]. The deregulated subcellular localization of p53 presents an extremely interesting drug target. For example, the inactivation of PARC by RNAi results in the restoration of nuclear p53 and restores the p53-mediated response to stress [36;37]. Nutlins are cis-imidazoline analogs that target the interaction between MDM2 and p53 preventing the degradation of p53 and allowing the p53 to accumulate. This accumulation enables p53 to induce senescence and apoptosis and is being tested to treat tumors that contain normal or wild type p53 [38]. In addition, all-trans-retinoic acid (RA) and retinoids inhibit the nuclear accumulation of p53 without substantially affecting its steady-state level. This suggests that nuclear import of p53 is an independent and active process that can be selectively regulated and targeted by RA to modulate chemoresistance [39]. However to ensure that the nuclear retention of p53 would be an effective treatment, patient screening is required to determine if their tumor contained wild-type p53. Despite this limitation, p53 remains a prime target for ongoing cancer drug development particularly in a personalized medicine context [40;41].

### FOXO proteins

Class O forkhead box (FOXO) proteins are a family of transcription factors (FOXO1, FOXO3a, FOXO4, and FOXO6) that control a number of essential cellular roles including cell cycle progression, metabolism, apoptosis, and stress resistance [42]. FOXO activity is regulated by subcellular translocation, DNA binding and protein degradation [43]. FOXO inhibition occurs through PI3K/Akt signalling whereas FOXO activation (via reactive oxygen signalling [ROS]) has been shown to converge in regard to the regulation of FOXO translocation. All FOXO proteins contain a non-classical bipartide NLS consisting of two clusters of arginine and lysine residues positioned on both sides of an Akt/14-3-3 binding motif in the C-terminus of the forkhead domain. Phosphorylation of the Akt introduces a negative charge in the basic stretch of residues that is required for NLS function, thereby inhibiting nuclear translocation of FOXO. In contrast the identity of the nuclear import receptors for FOXO proteins remains largely unknown. A recent publication suggests that distinct nuclear import pathways control ROS-dependent and insulin signalling-dependent nuclear localization of FOXO [44] and that ROS induce the formation of a disulfde bond between FOXO4 and transportin-1. This interaction is required for effcient nuclear localization of FOXO4 upon ROS signalling but not upon loss of insulin signalling [44]. FOXO proteins also contain a NES which mediates the CRM1-dependent nuclear export of non-DNA bound FOXO. The exposure of the FOXO-NES motif is facilitated by the binding of the chaperone protein 14-3-3 to FOXO proteins in the nucleus enabling their active export. 14-3-3 binding is regulated in response to growth signals by Akt and related SGK mediated phosphorylation of FOXO [45]. The activity of FOXO transcription factors is also controlled by other types of post-translational modifcations that include non-Akt/SGK-mediated phosphorylation, acetylation, methylation, glycosylation and monoubiquitination. The restoration of nuclear FOXO localization has been highlighted as a promising strategy to treat certain types of cancer and accordingly, the forced expression of nuclear FOXO has been shown to induce apoptosis in a wide range of cancer cells including prostate, and breast cancers as well as in malignant melanoma [46-51].

While it is highly tempting to therapeutically inhibit PI3K/Akt (that is constitutively active in many cancers) driving the accumulation of nuclear FOXO [52], a recent, important study revealed that in the presence of high nuclear β-catenin, that the activation of FOXO3a by PI3K or Akt inhibitors induced metastasis rather than mediating a pro-apoptotic anti-tumor response as would have been predicted [53]. This raises the hypothesis that the β-catenin status within patient carcinomas must be evaluated prior to deciding on this course of treatment. This evaluation would be essential for both the provision of a safer and more effective personalized treatment of colon cancer. Importantly, pharmaceutically strategies to induce nuclear exclusion of β-catenin might signifcantly improve clinical outcomes in these patients. Regardless, while there are some limitations, the FOXO family of proteins remains a viable and highly investigated anti-cancer target.

### p27

The cyclin-dependent kinase (CDK) inhibitor p27 displays aberrant subcellular localization in many cancers. In normal cells, p27 is localized in the nucleus where it binds to and inhibits CDK2, an activator of E2F1 transcription factors, promoting DNA replication [54;55]. To export p27 from the nucleus (via CRM1), p27 phosphorylated at serine 10 is required. Following this modifcation, p27 is exported into the cytoplasm and degraded by the cullin-RING ubiquitin ligase CRL1^SKP2^. The constitutive activation of Akt promotes this post-translational modifcation and directs the mis-localisation of p27 into the cytoplasm [56]. High levels of (cytoplasmic) phosphorylated serine 10-p27 have also been observed in glioma patients and correlated with both grade and clinical prognosis. In other carcinomas such as oesophagus, thyroid, colon and p27-positive breast carcinomas, p27 localization is also predominantly cytoplasmic thus allowing CDK2 to activate E2F1. This activation promotes cell-cycle progression driving tumorigenesis. To compound this further, Akt-mediated phosphorylation of the NLS within p27 also inhibits nuclear import further promoting the cytoplasmic sequestration of p27 [57], in a manner similar to the FOXO transcription factors discussed above.

p27 is an interesting therapeutic target as a number of compounds are being screened and evaluated [58] with particular attention focused on compounds that directly disrupt the binding of phosphorylated p27 to the CRL1 substrate receptor (SKPins) [59]. This approach offers considerably higher target specifcity compared to broad proteasome inhibitors such as Bortezomib or Carflzomib that are currently administered in the clinic that are associated with signifcant toxicity and patient side effects. Clearly before administering these compounds, aberrant p27 localization would need to be observed and thus underpins a personalized treatment platform for colon, glioma and breast cancer.

### BRCA1

The breast cancer susceptibility gene 1 (BRCA1) product has multiple roles within the cell, interacting with tumor suppressors, DNA repair proteins and cell cycle regulators [60]. Due to the signifcant size of the protein (220 kDa) BRCA1 can only enter the nucleus via active transport. BRCA1 contains two NLS within a single exon [61] and is transported into the nucleus by the importin-α/ß pathway [62]. BRCA1 can also form a protein-protein complex with the BRCA1-associated RING domain protein 1 (BARD1) and following this interaction can also accumulate within the nucleus without requiring the BRCA1 NLS [63]. While nuclear accumulation is clearly evident BRCA1 also undergoes receptor-mediated nuclear export [64]. BRCA1 has a Rev-like NES that is classically bound by CRM1 and more interestingly BARD1 has been shown to mask the BRCA1 NES, indicating that BARD1 can regulate both the nuclear import and export of BRCA1 [65]. Due to the extremely complex regulation of BRCA1 subcellular localization, it is not surprising that a plethora of mutations were characterized from breast cancer patients resulting in either the loss or gain of BRCA1 function.

PARP (poly (ADP-ribose) polymerase) inhibitors have been shown to be effective in treating BRCA1-mutated tumors. The therapeutic exclusion of BRCA1 from the nucleus could signifcantly sensitize cancers to PARP inhibitors, increasing the effectiveness of these compounds and/or reducing the effective concentration required, minimizing patient toxicity. In the context of a personalized medicine approach this could enable PARP inhibitor regimes to be used to treat tumors that display non-mutated, nuclear BRCA1 [64;66;67]. Given the increasing emergence of drug resistant breast cancers and the molecular profling already conducted from breast cancer biopsies, the incorporation of BRCA1 status/localization could offer signifcant hope to patients who fail to respond to conventional therapeutics.

### β-Catenin

ß-catenin has been shown to be a key component of the Wnt signalling pathway. Most cases of colon cancer are initiated by the nuclear accumulation of the β-catenin protein following mutation or due to the inactivation of the adenomatous polyposis coli (APC) tumor suppressor that regulates β-catenin stability. In the absence of Wnt signalling, β-catenin is rapidly phosphorylated by APC and glycogen synthase kinase 3 beta (GSK-3β). Phsophorylated β-catenin is quickly degraded in an ubiquitin- preoteosome-dependent manner allowing APC to maintain a low level of β-catenin within the cell. Wnt binding to Frizzled (Fz) and Lrp5/6 inhibits the APC complex which allows the β-catenin protein to accumulate and enter the nucleus, driving β-catenin-dependent gene expression [68]. Within the literature there remains no consensus regarding the mechanism of β-catenin subcellular localization although it is known that nuclear import is independent of the NLS/importin machinery. Protein structure and sequence studies demonstrated that β-catenin is a close relative of importins and can directly interact with the NPC [69]. β-catenin can also be transported back into the cytoplasm bound to Axin (a negative regulator of the Wnt signalling pathway), independent of its degradation role [70] or when in a protein complex with APC, migrating from the cytoplasm and the nucleus [71].

Given the widespread anomalous Wnt mediated signalling in cancer, targeting the nuclear localization of β-catenin represents a very attractive therapeutic strategy. As β-catenin does not rely on the classic nuclear import and export pathways, it presents a unique opportunity to specifcally interfere with its nucleo-cytoplasmic transport without signifcantly affecting non-transformed cells. Therapeutic molecules that mediate the specifc nuclear exclusion of β-catenin together with companion diagnostic tests to identify patients who may beneft from this treatment could lead to major breakthroughs in colon cancer treatment and disease management.

### APC

As mentioned above, the tumor suppressor APC regulates many cellular functions and promotes the degradation of β-catenin. In non transformed cell, APC is distributed evenly between the nucleus and cytoplasm with no signifcant accumulation in either compartment. APC itself is mutated in approximately 80% of all colon cancer patients [72] with the most common mutation resulting in a stable, truncated form of the protein that accumulates in the nucleus. The APC protein contains multiple NLS and NES signals and has been shown to shuttle between the nucleus and cytoplasm. It has been reported that an APC protein that lacks a functional NLS or NES signals fails to effectively down regulate nuclear β-catenin levels and this is also observed in cells that express wild-type APC that have nuclear export blocked [73].

Like β-catenin, APC is an attractive therapeutic target [74], particularly in regard to retaining or trapping APC within the cytoplasm. A complementary approach with compounds that mediate β-catenin nuclear exclusion could signifcantly improve the clinical outcome of colon cancer patients as targeting two deregulated molecular events could result in a highly cancer specifc treatment regime with a reduced possibility of drug resistance.

### NF-κB

The transcriptional activator, nuclear factor-κB (NF-κB), has been widely described in tumorigenesis and chemotherapy resistance. In non-cancerous cells, NF-κB forms a complex with the inhibitor IκB, which masks the NLS on NF-κB and prevents translocation of NF-κB to the nucleus. In non-transformed cells, IκB is phosphorylated and degraded by the proteasome revealing the NF-κB NLS enabling nuclear import [75]. In order to direct NF-κB into the cytoplasm, p300-mediated acetylation of NF-κB occurs, preventing assembly with IκB facilitating CRM1-dependent nuclear export via the NES presentation by NF-κB [76]. NF-κB is predominantly localized inside the nucleus in many cancers (for example Hodgkin's lymphoma, childhood acute lymphoblastic leukaemia, breast, colon and pancreatic cancers). This nuclear mis-localization can be attributed to defective IκB activity, hyperactivation of upstream kinases that result in IκB phosphorylation and degradation, or improper aceylation by p300 [76]. NF-κB contains classical NLS motifs and is imported into the nucleus via the importin α/β pathway. Since the nuclear localization of NF-κB subunits is a hallmark of a constitutively activated pathway and that this has been demonstrated to be essential for several types of human tumors, the inhibition of NF-κB nuclear import could prove highly benefcial for therapy against these cancers [77].

### Nucleophosmin

Nucleophosmin is a multifunctional protein that regulates ribosome biogenesis, RNA transcription, histone assembly and DNA repair [78]. This diverse range of roles refects its function as either an oncoprotein or tumor suppressor. Nucleophosmin is localized in the cytoplasm following apoptotic stimuli [79;80] and mediates this cellular response by regulating Bax activation. Nucleophosmin redistribution within the cell occurs prior to Bax translocation to the mitochondria. Nucleophosmin contains a NLS, a NES as well as a number of phosphorylation sites which control its cellular localization [81;82]. Despite the NES motifs, nucleophosmin remains localized within the nucleus indicating that under normal cellular conditions, its nuclear import is dominant over export, a process regulated by the Ran-CRM1 pathway [83]. Strikingly in acute myeloid leukaemia (with normal karyotype) there is aberrant nucleophosmin localization in the cytoplasm of cancer cells [84]. Molecular analysis of this cancer revealed that this was the result of an additional NES motif following a frame shift mutation, dramatically enhancing CRM1-dependent export [85].

Nucleophosmin is an interesting drug target, specifcally retargeting it back into the nucleus to prevent its aberrant cytoplasmic accumulation. Once within the nucleus it could, in principle drive Bax translocation. This restoration would be highly effective if used in combination with compounds that activate Bax which have recently been identifed and muted for potential clinical trial [86]. In a personalized medicine context, clinical samples would need to be evaluated for both aberrant nucleophosmin localization and the presence of Bax to mediate the cellular response following the retention of nucleophosmin within the nucleus.

### Survivin

Survivin is a member of the inhibitor of apoptosis protein (IAP) family that when it is localized within the nucleus, has a role in mitosis. In contrast, the cytoplasmic and mitochondrial localized survivin protein mediates an anti-apoptotic function [87-89]. The subcellular localization of survivin is CRM1/NES–dependent. When the survivin-NES is mutated, trapping survivin within the nucleus, proper cell division does not occur and the anti-apoptotic function of survivin is lost [90]. Many cancers (for example lung, colon, pancreas, prostate and breast), display an almost ubiquitous over expression of survivin compared to normal tissue correlating with clinical phenotype, resistance to therapy, and accelerated disease relapse [91;92]. Unlike within normal cells, survivin localization in cancer cells is at the inner mitochondrial membrane [88]. In non-transformed cells in response to an apoptotic stimulus, survivin is directed from the mitochondria to the cytosol where it inhibits apoptosis [88]. This function is dependent on the phosphorylation of survivin at residue Ser-20. This modifcation occurs in the cytosol but not in the mitochondria and it is this differential phosphorylation that regulates tumor cell apoptosis, modulating the interaction of survivin with the X-linked inhibitor of apoptosis protein (XIAP).

Survivin is an interesting target [93] particularly when one considers restoring or targeting the subcellular localization of proteins. Trapping survivin within the nucleus would ablate the anti-apoptotic function of the protein, signifcantly sensitizing the cancer to various chemotherapeutic regimes; however this could support the mitotic functions of the protein, driving cell division. Conversely, preventing survivin from nuclear entry would remove the pro-division functions but would still retain the anti-apoptotic function(s).

### Retinoblastoma (Rb)

The retinoblastoma (Rb) family of proteins play a crucial role regulating the cell cycle controlling the G_1_-S phase transition functioning as a potent tumor suppressor. The inactivation of pRB not only allows inappropriate proliferation but also undermines mitotic fdelity that results in genome instability and ploidy changes. Retinoblastoma defcient cells have an altered regulation of the G_1_ checkpoint and altered levels of autophagy, apoptosis, angiogenesis, and metastatic potential [94]. During the cell cycle, pRb-E2F forms a protein-protein complex that prevents transcription of E2F-regulated genes required for cell cycle progression [95;96]. This interaction with E2F is dependent upon pRb remaining non-phosphorylated [97]. Once phosphorylated by cyclinD1/CDK, pRb is targeted to the cytoplasm allowing G_1_ progression. Given this crucial inhibitory role, it is not surprising that Rb inactivation is a hallmark of many cancers.

The potency of Rb makes it an extremely attractive therapeutic target. Compounds that trap Rb within the nucleus of cancer cells could have a signifcant impact on a broad range of cancers, as these defects could represent an ‘Achilles heel’ that can be specifcally enhanced in tumor cells. The nuclear retention of pRb could stabilize the cancer genome and inhibit tumor evolution, effects that that could complement and dramatically enhance the potency of traditional therapeutics. There is a however a possibility that trapping Rb within the nucleus could prove ineffective due to aberrant, constitutive cyclin D1 activity (discussed below) driving tumorgenesis.

### Cyclin D1

Cyclin D1 is a member of the cyclin family of proteins that function as regulators of Cyclin-dependent kinases (CDKs). Cyclin D1 forms a complex with CDK4 and CDK6. Upon entry into the nucleus, active cyclin D1/CDK4 and Cyclin D1/CDK6 complexes direct the phosphorylation of Rb. As described above, phosphorylated Rb protein is exported into the cytoplasm and releases the E2F transcription factor promoting the transcription of S-phase genes driving cell cycle progression. Due to the potency of cyclin D1, the level of this protein is very carefully regulated within the cell. Prior to the poly-ubiquitination of cyclin D1 there is the requirement for phosphorylation at Thr-286 by GSK-3β. GSK-3β is retained within the cytoplasm of a cell during G_1_ but enters the nucleus during S-phase allowing access to the cyclin D1/CDK complex. Upon phosphorylation at Thr-286 cyclin D1 can then be bound by CRM1 and shuttled into the cytoplasm and degraded [55].

Given the role of cyclin D1 to promote the cell cycle it was surprising that frst, the amplifcation of the cyclin D1 locus is rarely observed in cancers with over expression of cyclin D1 and that second, the enforced over expression of cyclin D1 is not the transforming property of cyclin D1 [98]. Rather it is the nuclear retention of cyclin D1/CDK kinase that is the cancer predisposing mechanism [99]. A novel cyclin D1 isoform (cyclin D1b) was identifed that lacks both the GSK-3β and CRM1 binding site and is constitutively nuclear [100]. This isoform is still capable of binding and activating CDK4 and is also able to dissociate pRb (ensuring its cytoplasmic localization and subsequent degradation) promoting the cell to divide. Another cyclin D1 mutation is a proline-serine/threone substitution at residue 287 (preventing Thr-286 phosphorylation by GSK-3β) producing a constitutively nuclear mutant [101]. In addition to these splice isoforms and direct mutations, the signalling pathways (Ras-PI3K-Akt-GSK-3β) that regulate cyclinD1 localization are commonly deregulated in cancer. In light of these reports, it is thought that tumor suppression breaks down when the cell is no longer capable of shuttling cyclin D1 from the nucleus. As nuclear cyclin D1 is oncogenic, preventing the nuclear accumulation of this protein in patients with tumors that contain high levels of nuclear cyclin D1 could signifcantly improve clinical prognosis.

### Therapeutic Targeting of the Nucleo-cytoplasmic transport

**Figure 1:**
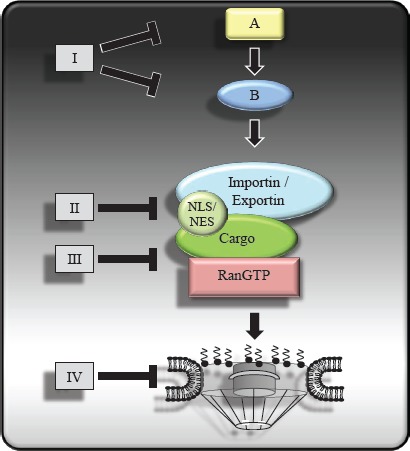
Potential therapeutic targets at different level of the nucleo-cytoplasmic transport process Therapeutic agents such as small molecules or biologics targeting nucleo-cytoplasmic transport of oncoproteins or tumor suppressor proteins can interfere with upstream regulatory components (I), the interaction between cargo proteins and the transport receptors (II), the interaction between the transport receptors and the Ran regulators (III) and the NPC (IV).

**Figure 2:**
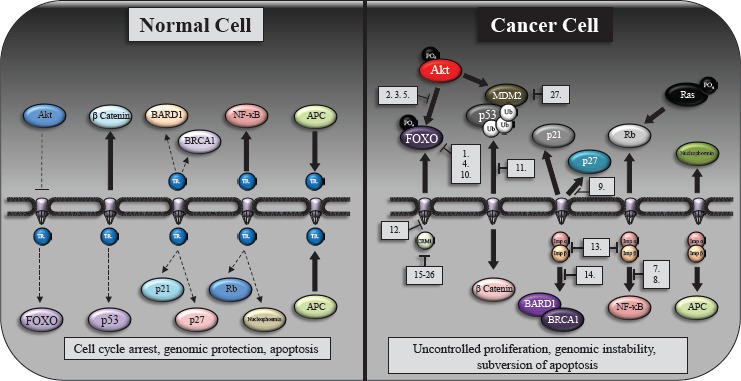
The subcellular distribution of oncogenes and tumor suppressors in normal cells and their redistribution following transformation A schematic indicating the subcellular localization of key protein in normal (left hand side) and cancer cells (right hand side). Transport receptors (TR) are broadly indicated within normal cells however we highlight specifc nuclear import/export proteins and indicate specifc agents (numbered 1-26 and listed in Table 2) that can target the aberrant protein localization within a number of cancers. As can be seen in the fgure, signifcant components of these aberrant pathways (particularly Ras, Rb and β-catenin subcellular localization) have yet to be targeted

Therapeutically targeting the nuclear transport of proteins is a very interesting option. Our understanding of this process has enabled specifc regions of the transport process to be targeted, indicated in Figure 1. The restoration of nucleo-cytoplasmic homeostasis correcting the disrupted physiological localization of tumor suppressors (e.g. p53, FOXO, p27, APC) or directing the mislocalization of proteins to inactivate oncoproteins (e.g. β-catenin, NF-κB, or Survivin) is currently being tested. These approaches are limited as normal and cancer cells share similar core components of the export and import apparatus. However, recent progress in understanding the nucleo-cytoplasmic communication process and its role in tumor formation and progression provides a previously unrecognized therapeutic avenue [102]. The cycles of nuclear import and export are governed by at least six types of protein–protein interactions, but only one enzymatic reaction occurs, namely the hydrolysis of GTP by RanGTPase. Our summarized Table 2 provides an overview of agents that are capable of interfering with nucleo-cytoplasmic traffcking and Figure 2 shows how the aberrant subcellular localization of proteins in cancer can be exploited by these agents as well as highlighting proteins/pathways yet to be specifcally targetted.

**Table 2:** Examples of agents capable of interfering with protein trafficking

Number	Agent	Type of Regulation	Primary target	Translocation effect	Reference
1).	Trifuoperazine hydrochloride	upstream regulation	Dopamine receptor	Nuclear FOXO localization	[107]
2).	W13	upstream regulation	Ca2+/Calmodulin	Nuclear FOXO3a localization	[106]
3).	ETP-45648	upstream regulation	PI3K	Nuclear FOXO localization	[125]
4).	Vinblastine	upstream regulation	Tubulin	Nuclear FOXO localization	[106]
5).	Akt inhibitor X	upstream regulation	Akt	Nuclear FOXO localization	[154]
6).	INCAs	upstream regulation	Calcineurin	Cytoplasmic NFAT	[109]
7).	BAY 11-7082	upstream regulation	IκB kinase [IKK]	Cytoplasmic NF-κB	[110]
8).	CHS828	upstream regulation	IKK	Cytoplasmic NF-κB	[155]
9).	SMIP001/004	upstream regulation	unknown	Nuclear p27KIP localization	[59]
10).	Resveratrol	upstream regulation	Sirt1	Nuclear FoxO1	[156]
11).	Elliticine	upstream regulation	unknown	Increased nuclear p53 localization	[157]
12).	WGA	NPC	GlcNAc	Unspecifc nuclear exclusion	[114]
13).	cSN50 peptide	Transport Receptor/Cargo	Importin-α	Cytoplasmic NF-κB, NFAT, AP1, STAT1	[119]
14).	bimax1/2 peptide	Transport Receptor/Cargo	importin-α	Cytoplasmic SV40, NP	[120]
15).	Leptomycin B	Transport Receptor/Cargo	CRM1	nuclear NES containing proteins	[27]
16).	Anguinomycins	Transport Receptor/Cargo	CRM1	nuclear NES containing proteins	[126]
17).	Goniothalamin	Transport Receptor/Cargo	CRM1	nuclear NES containing proteins	[158]
18).	Ratjadone	Transport Receptor/Cargo	CRM1	nuclear NES containing proteins	[128]
19).	Valtrate	Transport Receptor/Cargo	CRM1	nuclear NES containing proteins	[130]
20).	Acetoxychavicol acetate	Transport Receptor/Cargo	CRM1	nuclear NES containing proteins	[159]
21).	15d-PGJ2	Transport Receptor/Cargo	CRM1	nuclear NES containing proteins	[160]
22).	Peumusolide A	unknown	CRM1	Nuclear ERK	[159]
23).	PKF050-638	Transport Receptor/Cargo	CRM1	nuclear NES containing proteins	[134]
24).	SINE	Transport Receptor/Cargo	CRM1	nuclear NES containing proteins	[2]
25).	KOS-2464	Transport Receptor/Cargo	CRM1	nuclear NES containing proteins	[135]
26).	CBS9106	Transport Receptor/Cargo	CRM1	nuclear NES containing proteins	[136]

### Upstream manipulation of nucleo-cytoplasmic shuttling

As the interaction of cargo proteins with transport receptors has been shown to be regulated by dynamic exposure to transport signals, the manipulation of the upstream regulatory processes has the potential to interfere with the location and function in a cargo-specifc manner. Striking examples for upstream manipulation of nucleo-cytoplasmic shuttling are compounds that interfere with the subcellular localization of FOXO transcription factors. Small molecules known to inhibit different elements of the PI3K/Akt signaling pathway such as broad and isoform-specifc PI3K inhibitors, Akt inhibitors and PDK1 inhibitors decrease the activity of the downstream effector kinase Akt releasing FOXO proteins from their 14-3-3 anchors [103-105]. In addition, Ca^2+^/Calmodulin inhibitors that descrease Akt phosphorylation also leads to the accumulation of FOXO reporter proteins in the nucleus of U2OS cells [106]. As FOXO proteins are inactivated in many human cancers through alterations in their subcellular localization the above mentioned agents hold promise to be effective anticancer drugs restoring the FOXO tumor suppressor functions. Interestingly, Trifuoperazine hydrochloride (TFP), an FDA-approved antipsychotic and antiemetic inhibits FOXO1 nuclear export (107) and restored sensitivity to AKT-driven erlotinib resistance in both cell culture and xenograft models of lung adenocarcinoma [108].

Additional examples of transcription factors whose subcellular localization can be infuenced by small chemical compounds include the cancer-relevant proteins NFAT and NF-κB. The immunodepressive drugs cyclosporin A and FK506 inhibit the phosphatase activity of calcineurin and, in turn, the dephosphorylation-mediated unmasking of the NLS in the nuclear factor of activated T cells (NFAT) transcription factor, preventing its nuclear import. More specific inhibitors of NFAT-calcineurin association (e.g. INCA) have been developed and were shown to inhibit nuclear NFAT localization [109]. Furthermore and consistent with its inhibitory effect on I-κBα phosphorylation and NF-κB activation BAY 11-7082 has been identified as a specific inhibitor of the nuclear import of NF-κB [110].

### Inhibition of nuclear transport by interfering with NPC

Although the nucleoporin/transport receptor interaction represents an obvious target to inhibit the nucleo-cytoplasmic transport, it has not been explored extensively [111;112]. A recent study identifed a peptidomimetic inhibitor of the importin-α/β mediated import that mimics the FXFG structure and might block the binding of importin β to nucleoporins [113]. Monoclonal antibodies directed against nucleoporins have been used successfully in rat liver nuclear envelopes to prevent cargo association with the NPC and block the translocation of proteins [114]. Nuclear import can also be blocked by wheat germ agglutinin (WGA), but the mechanism underlying treatment with this agent is not fully understood [115].

### Interference with the receptor/RanGTP interaction

The high nuclear concentration of RanGTP regulates the disassembly of the import complex and the formation of the export complex in the nucleus. Several recent studies report the identifcation of small molecule compounds capable of disrupting the binding of importin β to RanGTP. Affnity-based screening by confocal nanoscanning was used to identify several structurally related high affnity binders of importin β [116]. A pyrrole compound was shown to interfere with the interaction between importin β and RanGTP that blocked the nuclear import of GFP-NFAT in HeLa cells. This agent has been named Karyostatin 1A. Sonderholm *et al.* used FRET-based high throughput small molecule screen to investigate the protein-protein interaction between importin β and RanGTP. The authors reveal that importazole, a 2,4 diaminoquinazoline specifcally blocked importin-β-mediated nuclear import both in Xenopus egg extracts and cultured cells without the disruption of either transportin-mediated nuclear import or CRM1-mediated nuclear export [117].

### Competitive inhibition of cargo/receptor interaction

Competitive inhibition of the cargo protein and transport receptor represents an alternative approach to modulate the nucleo-cytoplasmic transport of proteins. Although the use of peptides carrying transport signals to displace the nuclear import or export of cargos from their transport receptors is an attractive therapeutic strategy, to date it has not yet been exploited in cancer therapy. Early work revealed that the cell-permeable peptide cSN50 carrying the NLS of the NF-kB inhibits not only the nuclear import of NF-kB, but also of other stress-responsive transcription factors including AP1, NFAT and STAT1 despite the presence of diverse NLS within these proteins [118;119]. Since these proteins have been reported to play important roles in several human malignancies this approach might prove to be an effcient anti-cancer therapeutic. Structure-based design has been used to generate the nuclear import inhibitor and M9M (which specifcally interferes with Kapβ2) prevented the nuclear entry of cargo proteins containing a NLS with a R/H/Kx(2–5)PY motif. Conversely, Kosugi *et al.* employed an experimental strategy to obtain general inhibitors of the importin-α/β pathway based on the activity profling of systematically mutated NLS peptide templates [10;120]. Using this method, the peptides bimax1 and bimax2 (which bind tightly to importin-α independently of importin-β) conferred resistance to cargo release activities in the nucleus. However, natural peptides are generally considered as poor drug candidates due to the ease that they are degraded by host cell enzymes, their low bioavailability, and their lack of specifcity.

### Small molecule nuclear export inhibitors (NEIs)

Of all the potential targets regarding nuclear-cytoplasmic transport, the export receptor CRM1 remains the best characterized therapeutic target. CRM1 is absolutely required for the nuclear export of many cancer related proteins such as p53, FOXO, Rb and Survivin.

A major breakthrough regarding nuclear export inhibition resulted from the identifcation of CRM1 as the cellular target of the nuclear export inhibitor leptomycin B (LMB) [121]. A seminal study by Kudo *et al.* showed that LMB binds CRM1 at the conserved cysteine residue corresponding to position 528 of the human protein and 529 of the *Schizosaccharomyces pombe* protein [122]. Alkylation of this cysteine residue by LMB in the hydrophobic groove of CRM1 disrupts CRM1 binding to the leucine-rich NES of cargo proteins thereby preventing the formation of the CRM1-cargo-RanGTP export complex. Interestingly the Cys 528 residue is not essential for CRM1 function and mutant forms of CRM1 that carry a serine in this position can substitute for wildtype CRM1 in mammalian cells [27]. However, a C258S substitution renders human cells completely resistant to LMB. LMB was the frst specifc inhibitor of nuclear export and is one of the striking examples of a small molecule compound capable of disrupting a protein-protein interaction, typically considered diffcult to target. Unfortunately, when tested therapeutically LMB (elactocin) was found to exhibit severe dose-limiting toxicity as profound anorexia and malaise in a Phase I clinical trial [123]. Although, there are currently no inhibitors of the nuclear export in clinical trials, several different natural products as well as semi-synthetic and synthetic compounds have been identifed and strategies are being developed to use them therapeutically. Several image-based high-content screening assays have been developed to assist the effcient identifcation of inhibitors of the nuclear export [124;125].

### Natural compound NEIs

The LMB analogs Anguinomycins have been reported to be potent antitumor agents that are active in the picomolar range and display selective cytotoxicity against transformed cells [126]. It is hypothesized that this selectivity is based on the interference with the pRb tumor suppressor. A truncated Anguinomycin analog was still capable of blocking nuclear export above 25nM [127] and based on this fnding, Goniothalamin, a related natural compound was identifed as a nuclear export inhibitor. The chemical structures of both compounds, the truncated Anguinomycin analog and Goniothalamin suggest that like LMB, they might irreversibly bind to Cys 528 of CRM1. Ratjadone, a compound structurally related to LMB, was obtained by Höfe *et al.* (1994) and shown to prevent nuclear export in an identical molecular mechanism to LMB [128]. Other natural compounds were identifed including valtrate and acetoxychavicol acetate (ACA) isolated from *Valeriana fauriei* and *Alpinia galangal*, respectively as compounds that covalently bind to Cys 528 of CRM1 [129;130]. Although valtrate and ACA are being developed as anti-viral compounds, they might be also useful as anti-cancer agents. Interestingly the same group recently reported two natural products that retained their inhibitory activity when in the presence of a biotinylated probe derived from LMB suggesting an unknown NES non-antagonistic mode of action [131]. A very recent study demonstrates the power of phenotypic cellular high content analysis of natural extracts to identify specifc inhibitors of the nuclear export [132]

### Semi-synthetic and synthetic NEIs

#### PKF050-638

Daelemans *et al.* identifed the synthetic small molecule PKF050-638 with a molecular mass of 292.7 kDa that reversibly disrupts CRM1-NES interaction in the micromolar range demonstrating strict structural requirements for its activity [133]. Structural studies on PKF050-638 revealed CRM1 inhibition and highlighted that the activity of these compounds was not solely correlated to the target cystine in CRM1, suggesting that more elements are involved [134].

#### Selective inhibitors of the nuclear export (SINE)

Based on various developed N-azolylacrylate analogs, the biopharmaceutical company Karyopharm Therapeutics developed orally active small molecule SINE compounds that irreversibly bound the Cys 528 residue within CRM1. These compounds are water soluble and have been shown to prevent the nuclear exit of HIV-Rev-GFP, p53, FOXO and topoisomerase IIa [2]. SINEs were effective against various colorectal cancer cell lines and were well tolerated in these studies [2].

#### KOS-2464

A medicinal chemical approach conducted by Kosan Biosciences (Bristol-Myers Squibb) based on modifying LMB yielded several semi-synthetic LMB derivatives that maintain the high potency of LMB, but are up to 16-fold less toxic than LMB *in vivo* [135]. The most potent derivative, KOS-2464 showed substantial effcacy in multiple mouse xenograft models and in contrast to cancer cells, KOS-2464 triggered cell cycle arrest, but not apoptosis in normal lung fbroblasts. Furthermore, the treatment of several p53 wild-type cell lines with KOS-2464 led to an up regulation and nuclear localization of p53 [135]. These data suggest that toxicity associated with LMB is linked to “off target” effects and provide proof of concept that nuclear export can be inhibited with manageable toxicities *in vivo*.

### Reversible nuclear export inhibitors

The high *in-vivo* toxicity of LMB might be due to its ability to covalently bind to Cys528 residue of CRM1 and irreversibly block the nuclear export of proteins. Therefore reversible nuclear export inhibitors hold signifcant promise to reduce toxic side effects and treat a range of cancers. Recently, scientists at the biopharmaceutical company CanBas have disclosed CBS9106, an orally-active synthetic small molecule which reversibly prevents CRM1-mediated nuclear export and is currently being developed as an anti-cancer agent in preclinical trials [136] although the mechanism of its reversible binding remains to be elucidated [137]. In addition, CBS9106 signifcantly reduces CRM1 protein levels without affecting *CRM1* mRNA expression. This effect could be reversed by adding bortezomib or LMB suggesting that CBS9106-mediated CRM1 inhibition results in proteasome-dependent CRM1 degradation. CBS9106 caused arrest of the cell cycle and induced apoptosis in a time- and dose-dependent manner for a broad spectrum of cancer cells while recovery was restored after removal of the drug. Oral administration of CBS9106 signifcantly suppressed tumor growth and prolongs survival in tumor bearing mice without a signifcant loss in body weight. A reduced level of CRM1 protein was also observed in tumor xenografts isolated from mice treated with CBS9106. Ongoing preclinical toxicology studies will determine whether this promising clinical candidate could be advanced to initial human clinical testing. Finally a recent patent application discloses a series of small molecule compounds as reversible inhibitors of the CRM1 driven export of Hiv-Ref-GFP and Survivin-GFP for the treatment or prophylaxis of cancer or viral diseases (EP 2 431 364 A1).

## CONCLUSIONS

The therapeutic targetting of the nucleo-cytoplasmic shuttling of macromolecules has emerged as a promising approach to treat human diseases. In particular, the subcellular localization of tumor suppressors and oncogenic proteins is tightly regulated and essential for their function. Because the deregulation of intracellular protein transport is crucially involved in the pathophysiology of a broad range of human cancers, it offers novel molecular targets at many different levels to normalize or to interfere therapeutically with protein localization. The cargo proteins, the transport receptors, the Ran regulators and the NPC have been proposed as targets for therapeutic intervention, and in some cases agents have been developed to successfully infuence subcellular protein distribution in disease states. The manipulation of the upstream regulatory processes has the potential to interfere with the location and function in a cargo-specifc manner. Importantly, genetic or epigenetic alterations of upstream regulatory pathways known to drive tumorigenesis often lead to the aberrant localization of downstream effector proteins. As these cargo proteins retain a wild type phenotype their physiological homeostasis might be restored by relocalization. Pharmacological strategies to interfere with the general cellular nucleo-cytoplasmic transport machinery for anticancer therapy are limited as normal and cancer cells share similar core components of the export and import apparatus. However desired specifcity could be achieved by several ways. Given the enormous complexity of the transport machinery and the number of involved proteins described in this review, a more comprehensive characterization of the nuclear import and export pathways for cancer-related proteins might reveal many specifc therapeutic targets such as specifc transport receptors or certain components of the NPC. At the moment, the only proteins of the transport apparatus being actively pursued as drug targets are CRM1 and importin-α/ß, which are involved in the transport of many cellular proteins including tumor suppressor and oncogenic proteins. Several agents have been developed against these targets, some of them with promising therapeutic windows. In a personalized medicine context drugs with pleiotropic targets such as CRM1 or importin-α/ß might prove to be extremely effective and signifcantly improve patient prognosis.

One of the major challenges of developing therapeutic agents to interfere with the nucleo-cytoplasmic transport is the predominance of protein-protein interactions over enzymatic reactions during the transport cycles. Targeting the fat and extended protein surfaces to disrupt protein-protein interactions using classical small molecule drug modalities has proven to be diffcult. Conversely, Leptomycin B-like compounds and several recent studies indicate that protein–protein interfaces might be more tractable than has been thought [138]. Importantly, emerging technologies that are being used to bridge the pharmacologic gap between small molecules and protein therapeutics, including peptide stapling and fragment-based drug discovery hold promise to traverse the critical surface features of proteins [139]. In addition to the already available CRM1 and importin-α/ß inhibitors, agents that specifcally interfere with alternative nuclear import and export pathways would be extremely useful as tool compounds for cell biology research have the potential to become therapeutic anti-cancer drugs at a time that they are vitally required [140].

## References

[1] Zanella F, Dos Santos NR, Link W (2013). Moving to the core: spatiotemporal analysis of Forkhead box O (FOXO) and nuclear factor-kappaB (NF-kappaB) nuclear translocation. Traffic.

[2] Turner JG, Dawson J, Sullivan DM (2012). Nuclear export of proteins and drug resistance in cancer. Biochem Pharmacol.

[3] Hung MC, Link W (2011). Protein localization in disease and therapy. J Cell Sci.

[4] Cronshaw JM, Krutchinsky AN, Zhang W, Chait BT, Matunis MJ (2002). Proteomic analysis of the mammalian nuclear pore complex. J Cell Biol.

[5] Kutay U, Guttinger S (2005). Leucine-rich nuclear-export signals: born to be weak. Trends Cell Biol.

[6] Xu S, Powers MA (2009). Nuclear pore proteins and cancer. Semin Cell Dev Biol.

[7] Ren Y, Seo HS, Blobel G, Hoelz A (2010). Structural and functional analysis of the interaction between the nucleoporin Nup98 and the mRNA export factor Rae1. Proc Natl Acad Sci U S A.

[8] Chin K, Devries S, Fridlyand J, Spellman P.T, Roydasgupta R, Kuo W.L, Lapuk A, Neve R.M, Qian Z, Ryder T, Chen F, Feiler H, Tokuyasu T, Kingsley C, Dairkee S, Meng Z (2006). Genomic and transcriptional aberrations linked to breast cancer pathophysiologies. Cancer Cell.

[9] Wong RW, Blobel G (2008). Cohesin subunit SMC1 associates with mitotic microtubules at the spindle pole. Proc Natl Acad Sci U S A.

[10] Moore D.J, Zienkiewicz J, Kendall P.L, Liu D, Liu X, Veach R.A, Collins R.D, Hawiger J (2010). In vivo islet protection by a nuclear import inhibitor in a mouse model of type 1 diabetes. PLoS One.

[11] Nakamura T, Largaespada D.A, Lee M.P, Johnson L.A, Ohyashiki K, Toyama K, Chen S.J, Willman C.L, Chen I.M, Feinberg A.P, Jenkins N.A, Copeland N.G, Shaughnessy J.D (1996). Fusion of the nucleoporin gene NUP98 to HOXA9 by the chromosome translocation t7;11.p15;p15. in human myeloid leukaemia. Nat Genet.

[12] Cooper CS, Park M, Blair DG, Tainsky MA, Huebner K, Croce CM, Vande Woude G.F (1984). Molecular cloning of a new transforming gene from a chemically transformed human cell line. Nature.

[13] Martinez N, Alonso A, Moragues MD, Ponton J, Schneider J (1999). The nuclear pore complex protein Nup88 is overexpressed in tumor cells. Cancer Res.

[14] Gould VE, Orucevic A, Zentgraf H, Gattuso P, Martinez N, Alonso A (2002). Nup88 karyoporin. in human malignant neoplasms and dysplasias: correlations of immunostaining of tissue sections, cytologic smears, and immunoblot analysis. Hum Pathol.

[15] Agudo D, Gomez-Esquer F, Martinez-Arribas F, Nunez-Villar MJ, Pollan M, Schneider J (2004). Nup88 mRNA overexpression is associated with high aggressiveness of breast cancer. Int J Cancer.

[16] Dahl E, Kristiansen G, Gottlob K, Klaman I, Ebner E, Hinzmann B (2006). Molecular profling of laser-microdissected matched tumor and normal breast tissue identifes karyopherin alpha2 as a potential novel prognostic marker in breast cancer. Clin Cancer Res.

[17] Kau TR, Silver PA (2003). Nuclear transport as a target for cell growth. Drug Discov Today.

[18] Noske A, Weichert W, Niesporek S, Roske A, Buckendahl AC, Koch I, Sehouli J, Dietel M, Denkert C (2008). Expression of the nuclear export protein chromosomal region maintenance/exportin 1/Xpo1 is a prognostic factor in human ovarian cancer. Cancer.

[19] Huang WY, Yue L, Qiu WS, Wang LW, Zhou XH, Sun YJ (2009). Prognostic value of CRM1 in pancreas cancer. Clin Invest Med.

[20] Yao Y, Dong Y, Lin F, Zhao H, Shen Z, Chen P, Sun Y.J, Tang L.N, Zheng S.E (2009). The expression of CRM1 is associated with prognosis in human osteosarcoma. Oncol Rep.

[21] Shen A, Wang Y, Zhao Y, Zou L, Sun L, Cheng C (2009). Expression of CRM1 in human gliomas and its signifcance in p27 expression and clinical prognosis. Neurosurgery.

[22] van der Watt PJ, Maske CP, Hendricks DT, Parker MI, Denny L, Govender D (2009). The Karyopherin proteins, Crm1 and Karyopherin beta1, are overexpressed in cervical cancer and are critical for cancer cell survival and proliferation. Int J Cancer.

[23] Kim IS, Kim DH, Han SM, Chin MU, Nam HJ, Cho HP (2000). Truncated form of importin alpha identifed in breast cancer cell inhibits nuclear import of p53. J Biol Chem.

[24] Behrens P, Brinkmann U, Fogt F, Wernert N, Wellmann A (2001). Implication of the proliferation and apoptosis associated CSE1L/CAS gene for breast cancer development. Anticancer Res.

[25] Wellmann A, Flemming P, Behrens P, Wuppermann K, Lang H, Oldhafer K, Pastan I, Brinkmann U (2001). High expression of the proliferation and apoptosis associated CSE1L/CAS gene in hepatitis and liver neoplasms: correlation with tumor progression. Int J Mol Med.

[26] Brinkmann U, Gallo M, Polymeropoulos MH, Pastan I (1996). The human CAS cellular apoptosis susceptibility. gene mapping on chromosome 20q13 is amplifed in BT474 breast cancer cells and part of aberrant chromosomes in breast and colon cancer cell lines. Genome Res.

[27] Kau TR, Way JC, Silver PA (2004). Nuclear transport and cancer: from mechanism to intervention. Nat Rev Cancer.

[28] Levine AJ, Greenbaum B (2012). The maintenance of epigenetic states by p53: the guardian of the epigenome. Oncotarget.

[29] Hollstein M, Sidransky D, Vogelstein B, Harris CC (1991). p53 mutations in human cancers. Science.

[30] Lu W, Pochampally R, Chen L, Traidej M, Wang Y, Chen J (2000). Nuclear exclusion of p53 in a subset of tumors requires MDM2 function. Oncogene.

[31] Tweddle DA, Malcolm AJ, Cole M, Pearson AD, Lunec J (2001). p53 cellular localization and function in neuroblastoma: evidence for defective G1. arrest despite WAF1 induction in MYCN-amplifed cells. Am J Pathol.

[32] Wei CL, Wu Q, Vega VB, Chiu KP, Ng P, Zhang T, Shahab A, Yong H.C, Fu Y, Weng Z, Liu J, Zhao X.D, Chew J.L, Lee Y.L, Kuznetsov V.A, Sung W.K, Miller L.D, Lim B, Liu E.T, Yu Q, Ng H.H, Ruan Y (2006). A global map of p53 transcription-factor binding sites in the human genome. Cell.

[33] Li M, Brooks CL, Wu-Baer F, Chen D, Baer R, Gu W (2003). Mono- versus polyubiquitination: differential control of p53 fate by Mdm2. Science.

[34] Pei D, Zhang Y, Zheng J (2012). Regulation of p53 a collaboration between Mdm2 and Mdmx. Oncotarget.

[35] Corvi R, Savelyeva L, Breit S, Wenzel A, Handgretinger R, Barak J, Oren M, Amler L, Schwab M (1995). Non-syntenic amplifcation of MDM2 and MYCN in human neuroblastoma. Oncogene.

[36] Moll UM, LaQuaglia M, Benard J, Riou G (1995). Wild-type p53 protein undergoes cytoplasmic sequestration in undifferentiated neuroblastomas but not in differentiated tumors. Proc Natl Acad Sci U S A.

[37] Nikolaev AY, Li M, Puskas N, Qin J, Gu W (2003). Parc: a cytoplasmic anchor for p53. Cell.

[38] Vassilev LT, Vu BT, Graves B, Carvajal D, Podlaski F, Filipovic Z, Kong N, Kammlott U, Lukacs C, Klein C, Fotouhi N, Liu E.A (2004). In vivo activation of the p53 pathway by small-molecule antagonists of MDM2. Science.

[39] Ronca F, Yee KS, Yu VC (1999). Retinoic acid confers resistance to p53-dependent apoptosis in SH-SY5Y neuroblastoma cells by modulating nuclear import of p53. J Biol Chem.

[40] Steelman LS, Martelli AM, Nicoletti F, McCubrey JA (2011). Exploiting p53 status to enhance effectiveness of chemotherapy by lowering associated toxicity. Oncotarget.

[41] Brown CJ, Lain S, Verma CS, Fersht AR, Lane DP (2009). Awakening guardian angels: drugging the p53 pathway. Nat Rev Cancer.

[42] Myatt SS, Lam EW (2007). The emerging roles of forkhead box (Fox) proteins in cancer. Nat Rev Cancer.

[43] Zanella F, Link W, Carnero A (2010). Understanding FOXO new views on old transcription factors. Curr Cancer Drug Targets.

[44] Putker M, Madl T, Vos HR, de RH, Visscher M, van den Berg MC, Kaplan M, Korswagen H.C, Boelens R, Vermeulen M, Burgering B.M, Dansen T.B (2013). Redox-dependent control of FOXO/DAF-16 by transportin-1. Mol Cell.

[45] Calnan DR, Brunet A (2008). The FoxO code. Oncogene.

[46] Yang H, Zhao R, Yang HY, Lee MH (2005). Constitutively active FOXO4 inhibits Akt activity regulates p27 Kip1 stability and suppresses HER2-mediated tumorigenicity. Oncogene.

[47] Segura MF, Hanniford D, Menendez S, Reavie L, Zou X, Alvarez-Diaz S, Zakrzewski J, Blochin E, Rose A, Bogunovic D, Polsky D, Wei J, Lee P, Belitskaya-Levy I, Bhardwaj N, Osman I, Hernando E (2009). Aberrant miR-182 expression promotes melanoma metastasis by repressing FOXO3 and microphthalmia-associated transcription factor. Proc Natl Acad Sci U S A.

[48] Hu MC, Lee DF, Xia W, Golfman LS, Ou-Yang F, Yang JY, Zou Y, Bao S, Hanada N, Saso H, Kobayashi R, Hung M.C (2004). IkappaB kinase promotes tumorigenesis through inhibition of forkhead FOXO3a. Cell.

[49] Hilmi C, Larribere L, Deckert M, Rocchi S, Giuliano S, Bille K, Ortonne J.P, Ballotti R, Bertolotto C (2008). Involvement of FKHRL1 in melanoma cell survival and death. Pigment Cell Melanoma Res.

[50] Zanella F, Renner O, Garcia B, Callejas S, Dopazo A, Peregrina S, Link W (2010). Human TRIB2 is a repressor of FOXO that contributes to the malignant phenotype of melanoma cells. Oncogene.

[51] Cornforth AN, Davis JS, Khanifar E, Nastiuk KL, Krolewski JJ (2008). FOXO3a mediates the androgen-dependent regulation of FLIP and contributes to TRAIL-induced apoptosis of LNCaP cells. Oncogene.

[52] Garrett JT, Chakrabarty A, Arteaga CL (2011). Will PI3K pathway inhibitors be effective as single agents in patients with cancer?. Oncotarget.

[53] Tenbaum SP, Ordonez-Moran P, Puig I, Chicote I, Arques O, Landolf S (2012). beta-catenin confers resistance to PI3K and AKT inhibitors and subverts FOXO3a to promote metastasis in colon cancer. Nat Med.

[54] Banin S, Moyal L, Shieh S, Taya Y, Anderson CW, Chessa L (1998). Enhanced phosphorylation of p53 by ATM in response to DNA damage. Science.

[55] Sherr CJ, Roberts JM (1999). CDK inhibitors: positive and negative regulators of G1-phase progression. Genes Dev.

[56] Blain SW, Massague J (2002). Breast cancer banishes p27 from nucleus. Nat Med.

[57] Shin I, Yakes FM, Rojo F, Shin NY, Bakin AV, Baselga J, Arteaga CL (2002). PKB/Akt mediates cell-cycle progression by phosphorylation of p27(Kip1) at threonine 157 and modulation of its cellular localization. Nat Med.

[58] Diersch S, Wenzel P, Szameitat M, Eser P, Paul MC, Seidler B (2013). Efemp1 and p27Kip1. modulate responsiveness of pancreatic cancer cells towards a dual PI3K/mTOR inhibitor in preclinical models. Oncotarget.

[59] Rico-Bautista E, Wolf DA (2012). Skipping Cancer: Small Molecule Inhibitors of SKP2-Mediated p27 Degradation. Chem Biol.

[60] Roy R, Chun J, Powell SN (2012). BRCA1 and BRCA2 different roles in a common pathway of genome protection. Nat Rev Cancer.

[61] Thakur S, Zhang HB, Peng Y, Le H, Carroll B, Ward T (1997). Localization of BRCA1 and a splice variant identifes the nuclear localization signal. Mol Cell Biol.

[62] Chen CF, Li S, Chen Y, Chen PL, Sharp ZD, Lee WH (1996). The nuclear localization sequences of the BRCA1 protein interact with the importin-alpha subunit of the nuclear transport signal receptor. J Biol Chem.

[63] Schuchner S, Tembe V, Rodriguez JA, Henderson BR (2005). Nuclear targeting and cell cycle regulatory function of human BARD1. J Biol Chem.

[64] Thompson ME (2010). BRCA1 16 years later: nuclear import and export processes. FEBS J.

[65] Fabbro M, Rodriguez JA, Baer R, Henderson BR (2002). BARD1 induces BRCA1 intranuclear foci formation by increasing RING-dependent BRCA1 nuclear import and inhibiting BRCA1 nuclear export. J Biol Chem.

[66] Fong PC, Boss DS, Yap TA, Tutt A, Wu P, Mergui-Roelvink M, Mortimer P, Swaisland H, Lau A, O'Connor M.J, Ashworth A, Carmichael J, Kaye SB, Schellens JH, de Bono JS (2009). Inhibition of poly(ADP-ribose) polymerase in tumors from BRCA mutation carriers. N Engl J Med.

[67] Zhang Y, Carr T, Dimtchev A, Zaer N, Dritschilo A, Jung M (2007). Attenuated DNA damage repair by trichostatin A through BRCA1 suppression. Radiat Res.

[68] Zeng X, Huang H, Tamai K, Zhang X, Harada Y, Yokota C (2008). Initiation of Wnt signaling: control of Wnt coreceptor Lrp6 phosphorylation/activation via frizzled dishevelled and axin functions. Development.

[69] Fagotto F, Gluck U, Gumbiner BM (1998). Nuclear localization signal-independent and importin/karyopherin-independent nuclear import of beta-catenin. Curr Biol.

[70] Cong F, Varmus H (2004). Nuclear-cytoplasmic shuttling of Axin regulates subcellular localization of beta-catenin. Proc Natl Acad Sci U S A.

[71] Rosin-Arbesfeld R, Townsley F, Bienz M (2000). The APC tumour suppressor has a nuclear export function. Nature.

[72] Powell SM, Zilz N, Beazer-Barclay Y, Bryan TM, Hamilton SR, Thibodeau SN (1992). APC mutations occur early during colorectal tumorigenesis. Nature.

[73] Neufeld KL, Zhang F, Cullen BR, White RL (2000). APC-mediated downregulation of beta-catenin activity involves nuclear sequestration and nuclear export. EMBO Rep.

[74] Sikandar S, Dizon D, Shen X, Li Z, Besterman J, Lipkin SM (2010). The class I HDAC inhibitor MGCD0103 induces cell cycle arrest and apoptosis in colon cancer initiating cells by upregulating Dickkopf-1 and non-canonical Wnt signaling. Oncotarget.

[75] Beg AA, Ruben SM, Scheinman RI, Haskill S, Rosen CA, Baldwin AS (1992). I kappa B interacts with the nuclear localization sequences of the subunits of NF-kappa B: a mechanism for cytoplasmic retention. Genes Dev.

[76] Chen LF, Greene WC (2003). Regulation of distinct biological activities of the NF-kappaB transcription factor complex by acetylation. J Mol Med (Berl).

[77] Zanella F, Dos Santos NR, Link W (2012). Moving to the Core: Spatiotemporal Analysis of Forkhead Box O (FOXO) and Nuclear Factor-kappaB (NF-kappaB) Nuclear Translocation. Traffc.

[78] Lindenboim L, Borner C, Stein R (2011). Nuclear proteins acting on mitochondria. Biochim Biophys Acta.

[79] Lindenboim L, Blacher E, Borner C, Stein R (2010). Regulation of stress-induced nuclear protein redistribution: a new function of Bax and Bak uncoupled from Bcl-x(L). Cell Death Differ.

[80] Park KS, Han BG, Lee KH, Kim DS, Kim JM, Jeon H (2009). Depletion of nucleophosmin via transglutaminase 2 cross-linking increases drug resistance in cancer cells. Cancer Lett.

[81] Hingorani K, Szebeni A, Olson MO (2000). Mapping the functional domains of nucleolar protein B23. J Biol Chem.

[82] Szebeni A, Olson MO (1999). Nucleolar protein B23 has molecular chaperone activities. Protein Sci.

[83] Wang W, Budhu A, Forgues M, Wang XW (2005). Temporal and spatial control of nucleophosmin by the Ran-Crm1 complex in centrosome duplication. Nat Cell Biol.

[84] Falini B, Nicoletti I, Martelli MF, Mecucci C (2007). Acute myeloid leukemia carrying cytoplasmic/mutated nucleophosmin (NPMc+ AML): biologic and clinical features. Blood.

[85] Nakagawa M, Kameoka Y, Suzuki R (2005). Nucleophosmin in acute myelogenous leukemia. N Engl J Med.

[86] Gavathiotis E, Reyna DE, Bellairs JA, Leshchiner ES, Walensky LD (2012). Direct and selective small-molecule activation of proapoptotic BAX. Nat Chem Biol.

[87] Rodriguez JA, Span SW, Ferreira CG, Kruyt FA, Giaccone G (2002). CRM1-mediated nuclear export determines the cytoplasmic localization of the antiapoptotic protein Survivin. Exp Cell Res.

[88] Dohi T, Beltrami E, Wall NR, Plescia J, Altieri DC (2004). Mitochondrial survivin inhibits apoptosis and promotes tumorigenesis. J Clin Invest.

[89] Colnaghi R, Connell CM, Barrett RM, Wheatley SP (2006). Separating the anti-apoptotic and mitotic roles of survivin. J Biol Chem.

[90] Knauer SK, Kramer OH, Knosel T, Engels K, Rodel F, Kovacs AF, Dietmaier W, Klein-Hitpass L, Habtemichael N, Schweitzer A, Brieger J, Rodel C, Mann W, Petersen I, Heinzel T, Stauber RH (2007). Nuclear export is essential for the tumor-promoting activity of survivin. FASEB J.

[91] Ambrosini G, Adida C, Altieri DC (1997). A novel anti-apoptosis gene survivin expressed in cancer and lymphoma. Nat Med.

[92] Altieri DC (2003). Validating survivin as a cancer therapeutic target. Nat Rev Cancer.

[93] Yan S, Li Z, Thiele CJ (2013). Inhibition of STAT3 with orally active JAK inhibitor AZD1480 decreases tumor growth in Neuroblastoma and Pediatric Sarcomas In vitro and In vivo. Oncotarget.

[94] Burkhart DL, Sage J (200). Cellular mechanisms of tumour suppression by the retinoblastoma gene. Nat Rev Cancer.

[95] Zhu L, Harlow E, Dynlacht BD (1995). p107 uses a p21CIP1-related domain to bind cyclin/cdk2 and regulate interactions with E2F. Genes Dev.

[96] Zamanian M, La Thangue NB (1993). Transcriptional repression by the Rb-related protein p107. Mol Biol Cell.

[97] Mittnacht S (1998). Control of pRB phosphorylation. Curr Opin Genet Dev.

[98] Quelle DE, Ashmun RA, Shurtleff SA, Kato JY, Bar-Sagi D, Roussel MF, Sherr CJ (1993). Overexpression of mouse D-type cyclins accelerates G1 phase in rodent fbroblasts. Genes Dev.

[99] Alt JR, Cleveland JL, Hannink M, Diehl JA (2000). Phosphorylation-dependent regulation of cyclin D1 nuclear export and cyclin D1-dependent cellular transformation. Genes Dev.

[100] Lu F, Gladden AB, Diehl JA (2003). An alternatively spliced cyclin D1 isoform cyclin D1b is a nuclear oncogene. Cancer Res.

[101] Moreno-Bueno G, Rodriguez-Perales S, Sanchez-Estevez C, Hardisson D, Sarrio D, Prat J, Cigudosa JC, Matias-Guiu X, Palacios J (2003). Cyclin D1 gene (CCND1) mutations in endometrial cancer. Oncogene.

[102] Faustino RS, Nelson TJ, Terzic A, Perez-Terzic C (2007). Nuclear transport: target for therapy. Clin Pharmacol Ther.

[103] Zanella F, Rosado A, Garcia B, Carnero A, Link W (2009). Using multiplexed regulation of luciferase activity and GFP translocation to screen for FOXO modulators. BMC Cell Biol.

[104] Martinez GS, Hernandez AI, Varela C, Rodriguez-Aristegui S, Lorenzo M, Rodriguez A, Lorenzo M, Rodriguez A, Rivero V, Martin JI, Saluste CG, Ramos-Lima F, Cendon E, Cebrian D, Aguirre E, Gomez-Casero E, Albarran M (2012). Identifcation of ETP-46321 a potent and orally bioavailable PI3K alpha delta inhibitor. Bioorg Med Chem Lett.

[105] Link W, Oyarzabal J, Serelde BG, Albarran MI, Rabal O, Cebria A (2009). Chemical interrogation of FOXO3a nuclear translocation identifes potent and selective inhibitors of phosphoinositide 3-kinases. J Biol Chem.

[106] Zanella F, Rosado A, Garcia B, Carnero A, Link W (2008). Chemical genetic analysis of FOXO nuclear-cytoplasmic shuttling by using image-based cell screening. Chembiochem.

[107] Kau TR, Schroeder F, Ramaswamy S, Wojciechowski CL, Zhao JJ, Roberts TM (2003). A chemical genetic screen identifes inhibitors of regulated nuclear export of a Forkhead transcription factor in PTEN-defcient tumor cells. Cancer Cell.

[108] Sangodkar J, Dhawan NS, Melville H, Singh VJ, Yuan E, Rana H, Izadmehr S, Farrington C, Mazhar S, Katz S, Albano T, Arnovitz P, Okrent R, Ohlmeyer M, Galsky M, Burstein D, Zhang D, Politi K, Difeo A (2012). Targeting the FOXO1/KLF6 axis regulates EGFR signaling and treatment response. J Clin Invest.

[109] Roehrl MH, Kang S, Aramburu J, Wagner G, Rao A, Hogan PG (2004). Selective inhibition of calcineurin-NFAT signaling by blocking protein-protein interaction with small organic molecules. Proc Natl Acad Sci U S A.

[110] Kwon YJ, Genovesio A, Youl KN, Hi CK, Jung S, David-Watine B, Nehrbass U, Emans N (2007). High-content classifcation of nucleocytoplasmic import or export inhibitors. J Biomol Screen.

[111] Davis JR, Kakar M, Lim CS (2007). Controlling protein compartmentalization to overcome disease. Pharm Res.

[112] Chahine MN, Pierce GN (2009). Therapeutic targeting of nuclear protein import in pathological cell conditions. Pharmacol Rev.

[113] Ambrus G, Whitby LR, Singer EL, Trott O, Choi E, Olson AJ (2010). Small molecule peptidomimetic inhibitors of importin alpha/beta mediated nuclear transport. Bioorg Med Chem.

[114] Gasiorowski JZ, Dean DA (2003). Mechanisms of nuclear transport and interventions. Adv Drug Deliv Rev.

[115] Yoneda Y, Imamoto-Sonobe N, Yamaizumi M, Uchida T (1987). Reversible inhibition of protein import into the nucleus by wheat germ agglutinin injected into cultured cells. Exp Cell Res.

[116] Hintersteiner M, Ambrus G, Bednenko J, Schmied M, Knox AJ, Meisner NC, Gstach H, Seifert JM, Singer EL, Gerace L, Auer M (2010). Identifcation of a small molecule inhibitor of importin beta mediated nuclear import by confocal on-bead screening of tagged one-bead one-compound libraries. ACS Chem Biol.

[117] Soderholm JF, Bird SL, Kalab P, Sampathkumar Y, Hasegawa K, Uehara-Bingen M (2011). Importazole a small molecule inhibitor of the transport receptor importin-beta. ACS Chem Biol.

[118] Torgerson TR, Colosia AD, Donahue JP, Lin YZ, Hawiger J (1998). Regulation of NF-kappa B AP-1 NFAT and STAT1 nuclear import in T lymphocytes by noninvasive delivery of peptide carrying the nuclear localization sequence of NF-kappa B p50. J Immunol.

[119] Lin YZ, Yao SY, Veach RA, Torgerson TR, Hawiger J (1995). Inhibition of nuclear translocation of transcription factor NF-kappa B by a synthetic peptide containing a cell membrane-permeable motif and nuclear localization sequence. J Biol Chem.

[120] Kosugi S, Hasebe M, Entani T, Takayama S, Tomita M, Yanagawa H (2008). Design of peptide inhibitors for the importin alpha/beta nuclear import pathway by activity-based profling. Chem Biol.

[121] Stade K, Ford CS, Guthrie C, Weis K (1997). Exportin 1 (Crm1p) is an essential nuclear export factor. Cell.

[122] Kudo N, Matsumori N, Taoka H, Fujiwara D, Schreiner EP, Wolff B, Yoshida M, Horinouchi S (1999). Leptomycin B inactivates CRM1/exportin 1 by covalent modifcation at a cysteine residue in the central conserved region. Proc Natl Acad Sci U S A.

[123] Newlands ES, Rustin GJ, Brampton MH (1996). Phase I trial of elactocin. Br J Cancer.

[124] Zanella F, Rosado A, Blanco F, Henderson BR, Carnero A, Link W (2007). An HTS approach to screen for antagonists of the nuclear export machinery using high content cell-based assays. Assay Drug Dev Technol.

[125] Zanella F, Lorens JB, Link W (2010). High content screening: seeing is believing. Trends Biotechnol.

[126] Hayakawa Y, Sohda KY, Shin-Ya K, Hidaka T, Seto H (1995). Anguinomycins C and D new antitumor antibiotics with selective cytotoxicity against transformed cells. J Antibiot Tokyo.

[127] Bonazzi S, Eidam O, Guttinger S, Wach JY, Zemp I, Kutay U, Gademann K (2010). Anguinomycins and derivatives: total syntheses modeling and biological evaluation of the inhibition of nucleocytoplasmic transport. J Am Chem Soc.

[128] Koster M, Lykke-Andersen S, Elnakady YA, Gerth K, Washausen P, Hofe G, Sasse F, Kjems J, Hauser H (2003). Ratjadones inhibit nuclear export by blocking CRM1/exportin 1. Exp Cell Res.

[129] Tamura S, Shiomi A, Kaneko M, Ye Y, Yoshida M, Yoshikawa M, Kimura T, Kobayashi M, Murakami N (2009). New Rev-export inhibitor from Alpinia galanga and structure-activity relationship. Bioorg Med Chem Lett.

[130] Murakami N, Ye Y, Kawanishi M, Aoki S, Kudo N, Yoshida M, Nakayama EE, Shioda T, Kobayashi M (2002). New Rev-transport inhibitor with anti-HIV activity from Valerianae Radix. Bioorg Med Chem Lett.

[131] Tamura S, Kaneko M, Shiomi A, Yang GM, Yamaura T, Murakami N (2010). Unprecedented NES non-antagonistic inhibitor for nuclear export of Rev from Sida cordifolia. Bioorg Med Chem Lett.

[132] Cautain B, de PN, Murillo G, V Munozde EM, Gonzalez M, V Tormo JR, Link W (2013). High-Content Screening of Natural Products Reveals Novel Nuclear Export Inhibitors. J Biomol Screen.

[133] Daelemans D, Afonina E, Nilsson J, Werner G, Kjems J, De CE (2002). A synthetic HIV-1 Rev inhibitor interfering with the CRM1-mediated nuclear export. Proc Natl Acad Sci U S A.

[134] Van NT, Pannecouque C, Vanstreels E, Stevens M, Dehaen W, Daelemans D (2008). Inhibition of the CRM1-mediated nucleocytoplasmic transport by N-azolylacrylates: structure-activity relationship and mechanism of action. Bioorg Med Chem.

[135] Mutka SC, Yang WQ, Dong SD, Ward SL, Craig DA, Timmermans PB, Murli S (2009). Identifcation of nuclear export inhibitors with potent anticancer activity in vivo. Cancer Res.

[136] Sakakibara K, Saito N, Sato T, Suzuki A, Hasegawa Y, Friedman JM, Kufe DW, Vonhoff DD, Iwami T (2011). CBS9106 is a novel reversible oral CRM1 inhibitor with CRM1 degrading activity. Blood.

[137] Mao L, Yang Y (2013). Targeting the nuclear transport machinery by rational drug design. Curr Pharm Des.

[138] Wells JA, McClendon CL (2007). Reaching for high-hanging fruit in drug discovery at protein-protein interfaces. Nature.

[139] Verdine GL, Walensky LD (2007). The challenge of drugging undruggable targets in cancer: lessons learned from targeting BCL-2 family members. Clin Cancer Res.

[140] Blagosklonny MV (2012). Common drugs and treatments for cancer and age-related diseases: revitalizing answers to NCI's provocative questions. Oncotarget.

[141] Takenaka Y, Fukumori T, Yoshii T, Oka N, Inohara H, Kim HR (2004). Nuclear export of phosphorylated galectin-3 regulates its antiapoptotic activity in response to chemotherapeutic drugs. Mol Cell Biol.

[142] Fabbro M, Henderson BR (2003). Regulation of tumor suppressors by nuclear-cytoplasmic shuttling. Exp Cell Res.

[143] Rodriguez JA, Schuchner S, Au WW, Fabbro M, Henderson BR (2004). Nuclear-cytoplasmic shuttling of BARD1 contributes to its proapoptotic activity and is regulated by dimerization with BRCA1. Oncogene.

[144] Karin M, Cao Y, Greten FR, Li ZW (2002). NF-kappaB in cancer: from innocent bystander to major culprit. Nat Rev Cancer.

[145] Colombo E, Martinelli P, Zamponi R, Shing DC, Bonetti P, Luzi L, Volorio S, Bernard L, Pruneri G, Alcalay M (2006). Delocalization and destabilization of the Arf tumor suppressor by the leukemia-associated NPM mutant. Cancer Res.

[146] Besson A, Assoian RK, Roberts JM (2004). Regulation of the cytoskeleton: an oncogenic function for CDK inhibitors?. Nat Rev Cancer.

[147] Chu IM, Hengst L, Slingerland JM (2008). The Cdk inhibitor p27 in human cancer: prognostic potential and relevance to anticancer therapy. Nat Rev Cancer.

[148] Ito K, Liu Q, Salto-Tellez M, Yano T, Tada K, Ida H, Huang C, Shah N, Inoue M, Rajnakova A, Hiong KC, Peh BK, Han HC, Ito T, Teh M, Yeoh KG, Ito Y (2005). RUNX3 a novel tumor suppressor is frequently inactivated in gastric cancer by protein mislocalization. Cancer Res.

[149] Craig E, Zhang ZK, Davies KP, Kalpana GV (2002). A masked NES in INI1/hSNF5 mediates hCRM1-dependent nuclear export: implications for tumorigenesis. EMBO J.

[150] Mancini M, Toker A (2009). NFAT proteins: emerging roles in cancer progression. Nat Rev Cancer.

[151] Planchon SM, Waite KA, Eng C (2008). The nuclear affairs of PTEN. J Cell Sci.

[152] Vigneri P, Wang JY (2001). Induction of apoptosis in chronic myelogenous leukemia cells through nuclear entrapment of BCR-ABL tyrosine kinase. Nat Med.

[153] Welcker M, Larimore EA, Frappier L, Clurman BE (2011). Nucleolar targeting of the fbw7 ubiquitin ligase by a pseudosubstrate and glycogen synthase kinase 3. Mol Cell Biol.

[154] Rosado A, Zanella F, Garcia B, Carnero A, Link W (2008). A dual-color fuorescence-based platform to identify selective inhibitors of Akt signaling. PLoS One.

[155] Olsen LS, Hjarnaa PJ, Latini S, Holm PK, Larsson R, Bramm E (2004). Anticancer agent CHS 828 suppresses nuclear factor-kappa B activity in cancer cells through downregulation of IKK activity. Int J Cancer.

[156] Frescas D, Valenti L, Accili D (2005). Nuclear trapping of the forkhead transcription factor FoxO1 via Sirt-dependent deacetylation promotes expression of glucogenetic genes. J Biol Chem.

[157] Xu GW, Mawji IA, Macrae CJ, Koch CA, Datti A, Wrana JL, Dennis J.W, Schimmer A.D (2008). A high-content chemical screen identifes ellipticine as a modulator of p53 nuclear localization. Apoptosis.

[158] Wach JY, Guttinger S, Kutay U, Gademann K (2010). The cytotoxic styryl lactone goniothalamin is an inhibitor of nucleocytoplasmic transport. Bioorg Med Chem Lett.

[159] Tamura S, Tsuji K, Yongzhen P, Ohnishi-Kameyama M, Murakami N (2010). Six new acylated anthocyanins from red radish Raphanus sativus. Chem Pharm Bull (Tokyo).

[160] Hilliard M, Frohnert C, Spillner C, Marcone S, Nath A, Lampe T, Fitzgerald D. J, Kehlenbach R.H (2010). The anti-infammatory prostaglandin 15-deoxy-delta(12,14)-PGJ2 inhibits CRM1-dependent nuclear protein export. J Biol Chem.

